# Urban Homelessness in California: A Multicity Analysis of Structural Constraints and Policy Implementation

**DOI:** 10.3390/ijerph23040537

**Published:** 2026-04-21

**Authors:** Peter G. Kreysa

**Affiliations:** Department of Family and Consumer Sciences, California State University, Long Beach, CA 90840, USA; peter.kreysa@csulb.edu

**Keywords:** homelessness, California, housing policy, urban inequality, housing insecurity, behavioral health, governance

## Abstract

**Highlights:**

**Public health relevance—How does this work relate to a public health issue?**

- Homelessness in California’s largest cities is driven by structural determinants, such as housing scarcity, behavioral–health system gaps, and economic precarity, that directly shape population health and access to essential services.
- City-level variation in policy implementation reveals how local capacity and governance influence exposure to health risks, including unsheltered living, chronic illness, and barriers to care.

**Public health significance—Why is this work of significance to public health?**

- Understanding how seven major cities interpret and operationalize statewide homelessness policies provides critical insight into why health outcomes for unhoused residents differ across urban regions.
- The study demonstrates that public health systems cannot address homelessness effectively without accounting for the structural and policy environments that produce and sustain health inequities.

**Public health implications—What are the key implications or messages for practitioners, policy makers and/or researchers in public health?**

- Findings highlight the need for integrated, cross-sector strategies that align housing, behavioral health, and social-service systems to reduce unsheltered homelessness and improve health outcomes.
- Policymakers and practitioners can use city-level comparisons to identify scalable interventions, strengthen implementation capacity, and design more equitable public health responses across diverse urban contexts.

**Abstract:**

Across California, the seven largest cities—Los Angeles, San Diego, San Jose, San Francisco, Fresno, Sacramento, and Long Beach—carry a disproportionate share of the state’s homelessness crisis, even though they operate under the same statewide policy framework. Each city’s homelessness system reflects its own history, political climate, and housing market conditions, and this study shows that a common set of structural forces especially severe housing scarcity, fragmented behavioral–health systems, and uneven local capacity shapes homelessness across these urban areas while producing different outcomes on the ground. Drawing on multidisciplinary research, statewide policy analyses, and municipal data, the analysis compares how cities interpret and implement key interventions, including permanent supportive housing, interim shelter expansion, prevention strategies, and enforcement-oriented responses. The findings make clear that California’s homelessness crisis cannot be reduced to a single cause; instead, understanding it requires a systems-oriented perspective that accounts for the intertwined economic, social, and policy forces shaping conditions in each community. By situating city-level strategies within broader statewide patterns, the study identifies points of convergence and divergence, as well as persistent structural constraints that limit the effectiveness of current responses, underscoring the need for coordinated, scalable, and context-responsive policy solutions.

## 1. Introduction

Homelessness in California has reached unprecedented levels, reflecting the interaction of structural housing scarcity, behavioral–health system limitations, economic inequality, and governance fragmentation. Although the state has invested heavily in housing, services, and system reforms, outcomes vary widely across cities, underscoring the importance of local conditions in shaping system performance. Research consistently shows that homelessness is not the result of isolated factors but, rather, emerges from interdependent structural forces that operate differently across regions [1,2,3].

California’s seven largest cities—Los Angeles, San Diego, San Jose, San Francisco, Fresno, Sacramento, and Long Beach—offer unique opportunities to examine how statewide pressures manifest in distinct local contexts. These cities share common structural determinants, including rising rents, limited affordable-housing production, and insufficient behavioral–health capacity, yet differ in governance structures, service infrastructures, and political dynamics [4]. Understanding these differences is essential for interpreting variation in homelessness outcomes and for identifying policy strategies that are both evidence-based and context-specific.

This study provides a comparative analysis of homelessness systems across the seven cities, synthesizing structural, institutional, and policy factors that shape local patterns. By integrating statewide research with city-level evidence, the analysis highlights how housing markets, governance arrangements, and service capacity interact to produce divergent outcomes within a shared policy environment. The findings contribute to the literature by clarifying the limits of programmatic interventions in the absence of structural reforms and by identifying governance features associated with more effective system performance.

## 2. Literature Review

Research on homelessness in California shows that the crisis is structurally produced through the interaction of housing scarcity, behavioral–health system gaps, economic inequality, and local governance practices [1,3]. These forces shape both statewide patterns and city-level variation, producing distinct homelessness dynamics even within a shared policy environment. This section synthesizes the major themes in the literature that inform the comparative analysis.

### 2.1. Structural Housing Constraints

California’s chronic housing shortage remains the most widely cited driver of homelessness. Decades of underproduction, restrictive zoning, and high development costs have produced extremely low vacancy rates and rents that far exceed local wages, particularly in coastal metropolitan areas [3,4,5]. Research shows that homelessness rises predictably when median rents exceed roughly 32% of median income, a threshold surpassed in all major California cities [3]. Inland regions, such as Fresno and Sacramento, face lower housing costs but also lower wages and limited supportive-housing capacity, creating different but equally significant structural pressures [1,6]. Across the state, the mismatch between housing supply and demand constrains the effectiveness of downstream interventions, including rapid rehousing, vouchers, and interim housing.

### 2.2. Behavioral–Health System Limitations

Behavioral–health system gaps, including insufficient treatment beds, workforce shortages, and fragmented service pathways, are a consistent theme across the literature. While serious mental illness and substance use disorders do not independently cause homelessness, they interact with structural housing scarcity to increase vulnerability and complicate stabilization [1,3]. Deinstitutionalization without adequate community-based investment, combined with limited integration across mental-health, substance-use, and housing systems, has produced persistent bottlenecks in service delivery [3,7,8,9,10]. These gaps are especially consequential for individuals experiencing chronic homelessness, who require coordinated medical, behavioral–health, and social-service supports to maintain housing stability.

### 2.3. Governance Fragmentation and Local Implementation

California’s homelessness response is shaped by a complex governance environment in which responsibilities are distributed across state agencies, counties, cities, and nonprofit providers. Counties typically oversee behavioral–health systems, while cities control land use and many direct services. This fragmentation contributes to inconsistent implementation of Housing First principles, uneven service capacity, and variation in enforcement practices [1,5,11]. Comparative research highlights that governance structures significantly influence system performance: centralized models (e.g., Long Beach) tend to exhibit stronger coordination, while fragmented systems (e.g., Los Angeles, Sacramento) face persistent challenges in aligning housing, health, and social-service interventions [6,12,13].

### 2.4. Measurement and Definitions

Homelessness measurement in the United States relies primarily on the HUD Point-in-Time (PIT) count, which provides a standardized snapshot but is widely recognized as an undercount, particularly for individuals living in vehicles, remote encampments, or doubled-up situations [1,2]. California’s Homeless Data Integration System (HDIS) offers a broader view by aggregating administrative data, revealing that annual service contacts far exceed PIT estimates [1]. Differences in definitions across federal agencies (e.g., HUD vs. Department of Education) further complicate comparisons and obscure the scale of housing instability that precedes literal homelessness [3,8]. These measurement limitations have policy implications, as funding formulas often rely on PIT data.

### 2.5. Racial and Social Disparities

Racial disparities are among the most persistent features of homelessness in California. Black, Native American, and Pacific Islander residents experience homelessness at rates far exceeding their population share, reflecting long-standing inequities in housing access, incarceration, child-welfare involvement, and economic opportunity [1,3]. LGBTQ+ individuals, particularly transgender people and youth, face elevated risks due to discrimination, family rejection, and limited access to affirming services. Veterans, older adults, and youth exiting foster care also exhibit distinct vulnerabilities shaped by trauma, disability, and systemic disconnection [3]. These disparities underscore the need for equity-centered approaches that address structural racism and institutional barriers.

### 2.6. State-Level Policy Framework

California has undertaken extensive policy initiatives to address homelessness, including major investments in interim and permanent housing, behavioral–health integration, and local system accountability. Key programs include:

- Homeless Housing, Assistance and Prevention (HHAP): flexible block grants supporting shelter, housing, and services, with wide variation in local implementation [1,14].
- Project Homekey: rapid acquisition and conversion of hotels and motels into interim or permanent housing, widely recognized for speed and cost-effectiveness [15,16].
- Project Roomkey: non-congregate shelter for medically vulnerable individuals during COVID-19, highlighting both the potential and limits of hotel-based models [1].
- Behavioral–Health Initiatives: including Enhanced Care Management and Community Supports, designed to integrate housing and health services for individuals with complex needs [1].

Despite these investments, state-local tensions persist. Local control over land use limits the state’s ability to accelerate housing production, and variation in administrative capacity contributes to uneven implementation across jurisdictions. The absence of a statewide right-to-shelter mandate further contributes to California’s unusually high unsheltered rate [1,2,4].

### 2.7. Intervention Models

California’s homelessness response incorporates a range of intervention models aligned with Housing First principles. Permanent supportive housing (PSH) remains the most effective intervention for individuals experiencing chronic homelessness, though outcomes depend heavily on the availability of integrated behavioral–health services [1,3]. Rapid rehousing is effective for many households but is constrained in high-cost markets where rental units are scarce. Interim housing models, including Navigation Centers, tiny home communities, safe parking programs, and hotel conversions, provide safer alternatives to unsheltered living but face structural limits related to operating costs, staffing shortages, and limited pathways to permanent housing [1,15,17,18]. Enforcement-oriented approaches remain common despite evidence that they do not reduce homelessness and may exacerbate instability [11]. These statewide structural and institutional dynamics manifest differently across local contexts.

## 3. Comparative City Analysis

California’s seven largest cities operate within the same statewide policy framework, yet their homelessness systems vary widely in structure, capacity, and outcomes. These differences reflect the interaction of local housing markets, governance arrangements, service infrastructures, political dynamics, and demographic patterns. This section provides a comparative analysis of each city, examining how structural pressures and institutional choices shape local responses to homelessness. Viewed side by side, the cities’ operations reveal common challenges as well as distinct local paths that inform the cross-city synthesis and statewide implications that follow.

### 3.1. Los Angeles

Los Angeles has the largest homelessness population in California and one of the highest unsheltered rates in the nation, reflecting the interaction of extreme housing scarcity, high development costs, and a fragmented governance environment [1,2,12]. Severe shortages of affordable and supportive housing, combined with rising rents and limited behavioral–health capacity, contribute to persistent chronic homelessness and high levels of vehicular and encampment-based living [12,19]. These structural pressures shape the city’s system performance more than any single intervention or program.

Over the past decade, Los Angeles has pursued some of the state’s most ambitious homelessness initiatives, including large-scale investments in permanent supportive housing, expanded interim housing, and hotel conversions through Project Homekey. While these efforts have increased capacity, progress has been slowed by long development timelines, high per-unit costs, and bottlenecks in the supportive-housing pipeline. Interim housing models such as A Bridge Home have provided safer alternatives to unsheltered living, but throughput remains limited due to the scarcity of permanent housing options [12,15,20].

Governance complexity remains one of Los Angeles’s defining challenges. With 88 incorporated cities, a county government responsible for behavioral health, and numerous service providers operating across overlapping jurisdictions, coordination is difficult and often inconsistent. Public pressure to address visible encampments has contributed to periodic enforcement actions, creating tensions with Housing First principles and evidence-based practice [11]. Los Angeles illustrates how scale, structural scarcity, and governance fragmentation interact to constrain system performance, even in a region with substantial financial investment and political attention [1,11,12].

### 3.2. San Diego

San Diego faces rising homelessness driven by escalating rents, extremely low vacancy rates, and persistent gaps in behavioral–health and supportive-service capacity. Geographic dispersion across canyons, riverbeds, and peripheral areas complicates outreach and contributes to undercounting and inconsistent service engagement [1,21,22]. These structural pressures shape the city’s homelessness conditions more than any individual program or intervention.

The city has pursued a multifaceted response that blends Housing First principles with pragmatic adaptations to local constraints. San Diego has been one of the most active jurisdictions in leveraging Project Homekey to expand interim and permanent housing through hotel conversions, which have proven effective for individuals with medical or behavioral–health vulnerabilities [15,21]. Rapid rehousing and prevention programs show promise but remain constrained by high rents and limited landlord participation [17]. Multidisciplinary outreach teams have expanded engagement capacity, yet throughput remains limited as inflow continues to exceed exits into permanent housing [3,21].

Governance fragmentation and political pressure surrounding visible encampments shape implementation. While the city has expanded outreach and interim housing, periodic enforcement actions persist, creating tensions with Housing First principles and evidence-based practice. San Diego illustrates the challenges of implementing Housing First in a high-cost, low-vacancy environment where structural housing pressures outpace programmatic capacity [1,11]. Its experience contrasts with cities such as San Jose, where prevention infrastructure is more developed, and Fresno, where enforcement plays a more prominent role [23,24].

### 3.3. San Jose

San Jose has one of the highest per capita homelessness rates in the nation, shaped by extreme housing costs, limited rental availability, and rising levels of vehicular homelessness. Median rents far exceed local wages, making even modest economic shocks destabilizing for low-income households [4]. These structural pressures constrain the effectiveness of downstream interventions and contribute to persistent inflow into homelessness [2,4,18].

The region has pursued a broad portfolio of strategies that reflect strong alignment with Housing First and prevention-oriented approaches. Santa Clara County’s Measure A bond and Project Homekey acquisitions have expanded deeply affordable and supportive housing, while interim models such as tiny home communities and safe parking programs address the needs of individuals living in vehicles [15,25,26]. The countywide Homelessness Prevention System stands out as one of the most robust in the state, demonstrating unusually strong outcomes in reducing inflow through targeted financial assistance and legal support [27].

San Jose’s regional governance structure, which is anchored by Santa Clara County, supports strong coordination across behavioral health, prevention, and supportive services, yet also complicates accountability and performance monitoring [23]. Persistent behavioral–health workforce shortages and extreme housing scarcity continue to limit system throughput even as the region demonstrates some of the most innovative prevention and interim housing strategies in California [1,25]. San Jose illustrates the tension between strong program design and the broader structural forces that drive homelessness in high-growth metropolitan areas.

### 3.4. San Francisco

San Francisco exhibits one of the highest per capita homelessness rates in California, shaped by extreme housing scarcity, exceptionally high rents, limited land availability, and sustained inflow from individuals seeking economic opportunity or access to services [28]. The city’s homelessness landscape reflects long-standing structural pressures that outpace even substantial public investment, contributing to persistent unsheltered homelessness and high concentrations of individuals with complex behavioral–health needs [3,28,29].

San Francisco operates one of the largest permanent supportive housing portfolios in the state and has pioneered service-intensive interim housing models such as Navigation Centers, which provide low-barrier shelter and intensive case management [30]. These models demonstrate strong engagement and housing-retention outcomes, but face constraints related to high operating costs, limited throughput, and bottlenecks in the permanent-housing pipeline [1,31]. The city also invests heavily in harm-reduction and public-health initiatives, including syringe access, overdose prevention, and mobile crisis response [29,31,32].

Despite centralized governance, San Francisco faces persistent tensions between public pressure to address visible encampments and evidence-based practice. Periodic enforcement actions and legal disputes illustrate the difficulty of balancing harm-reduction approaches with political demands for encampment resolution [11,33]. San Francisco highlights the limits of service-intensive models in the absence of sufficient affordable and supportive housing and provides a useful contrast to cities such as Fresno, where enforcement plays a more prominent role, and San Jose, where prevention infrastructure is more developed.

### 3.5. Fresno

Fresno faces a distinct homelessness context shaped by regional poverty, limited behavioral–health infrastructure, and a long-standing reliance on enforcement-oriented approaches. Although housing costs are lower than in coastal cities, wages are also significantly lower and economic precarity plays a central role in driving homelessness. Geographic spread and limited public transportation further complicate outreach and service delivery, contributing to persistent unsheltered homelessness [1,3,24].

In recent years, Fresno has expanded low-barrier shelter capacity, participated in Project Homekey acquisitions, and piloted small-scale interim housing models to provide safer alternatives to encampments. However, service capacity, particularly behavioral health, remains limited, constraining the effectiveness of Housing First and permanent supportive housing models. Outreach teams face staffing shortages and limited treatment availability, creating bottlenecks in service engagement and housing stabilization [15,24,34].

Fresno illustrates how limited-service infrastructure and political emphasis on enforcement shape local homelessness responses. While the city has increased investment in housing and services, enforcement remains a prominent component of its approach, despite evidence that such strategies do not reduce homelessness and may exacerbate instability. Fresno provides an important contrast to cities such as San Francisco, where service-intensive models dominate, and San Jose, where prevention infrastructure is more developed.

### 3.6. Sacramento

Sacramento has experienced a sharp rise in homelessness driven by rapid population growth, rising rents, and limited affordable-housing production [35]. The region’s unsheltered rate remains among the highest in the state, reflecting both structural housing pressures and significant behavioral–health system gaps. Suburbanization and sprawling geography further complicate outreach, service coordination, and data collection [6].

The city has expanded interim housing through Safe Ground sites, congregate and non-congregate shelters, and Project Homekey acquisitions, which have increased capacity more quickly than traditional development models. Sacramento has also invested in behavioral–health services, including crisis stabilization units and mobile crisis teams, yet workforce shortages and fragmented service pathways continue to impede progress. Safe Ground sites illustrate a harm-reduction approach to encampment management, but face challenges related to limited capacity, neighborhood opposition, and insufficient pathways to permanent housing [6,10,36].

Governance fragmentation between the City of Sacramento, Sacramento County, and suburban jurisdictions remains one of the region’s most significant barriers. Coordination challenges particularly around behavioral–health responsibilities contribute to inconsistent implementation of Housing First principles and hinder the development of a cohesive regional strategy [6]. Sacramento occupies a middle position in the comparative landscape; it faces structural pressures similar to coastal cities but with fewer resources, less developed service infrastructure, and more pronounced governance challenges [1].

### 3.7. Long Beach

Long Beach faces rising homelessness driven by increasing rents, limited affordable-housing production, and regional economic pressures, yet benefits from a more centralized governance structure than neighboring Los Angeles. The city’s homelessness conditions reflects both coastal-level housing pressures and a more integrated service system, contributing to relatively stronger coordination across outreach, shelter, and housing programs [1,13,15].

Long Beach operates a comprehensive homelessness response anchored by the Multi-Service Center (MSC), which provides coordinated entry, case management, housing navigation, and access to behavioral–health and social-service supports. The city has expanded interim housing through congregate shelters, non-congregate hotel rooms, and specialized facilities for families, youth, and individuals with behavioral–health needs. Project Homekey acquisitions have increased non-congregate options and created new pathways to permanent supportive housing, though long-term sustainability depends on stable operating funds and expanded service capacity [13].

Despite stronger coordination, Long Beach faces significant structural constraints, including rising rents, insufficient behavioral–health capacity, and high rates of unsheltered homelessness. Public pressure to address visible encampments has led to periodic enforcement actions, creating tensions with Housing First principles [1,13]. Long Beach illustrates how centralized governance can enhance system performance even in the context of regional housing scarcity, offering a useful contrast to more fragmented systems such as Los Angeles and Sacramento.

## 4. Cross-City Synthesis

The experiences of California’s seven largest cities reveal both shared structural pressures and meaningful differences in local homelessness systems. Although each city operates within the same statewide policy environment, their paths diverge due to variations in housing markets, governance structures, service capacity, political dynamics, and demographic patterns. This synthesis identifies the major cross-cutting themes that emerge from the comparative analysis and highlights their implications for statewide policy design and local implementation.

### 4.1. Housing Scarcity Is the Dominant Structural Driver Across All Cities

Despite regional variation, every city faces a severe shortage of affordable housing. Coastal cities (Los Angeles, San Diego, San Jose, and San Francisco) experience the most acute pressures, with extremely low vacancy rates and rents that far exceed local wages [3,4,5]. These conditions undermine the effectiveness of rapid rehousing, prevention, and voucher-based programs, as households struggle to secure units even when financial assistance is available.

In inland cities, such as Fresno and Sacramento, housing costs are lower but still rising faster than incomes [1,6]. Economic difficulties, unemployment, and limited supportive-housing capacity play a more prominent role in driving homelessness [1,34]. Long Beach occupies a middle position, facing coastal-level housing pressures but benefiting from more centralized governance and a more integrated service system [13]. Across all cities, the structural mismatch between housing supply and demand limits the scalability of evidence-based interventions.

### 4.2. Behavioral Health System Gaps Are Universal and Deeply Constraining

Every city reports insufficient behavioral–health capacity, including shortages of clinicians, limited residential treatment beds, and fragmented service pathways [1,3,10]. These gaps are particularly consequential for individuals experiencing chronic homelessness, who often require integrated housing, medical, and behavioral–health support.

Los Angeles and San Francisco face the highest concentrations of individuals with complex behavioral–health needs [12,19,28]. San Diego and Sacramento struggle with geographic dispersion and limited treatment capacity [6,22], complicating access and continuity of care. Fresno experiences the most severe shortages relative to need, with minimal specialized infrastructure and limited provider availability [24]. San Jose and Long Beach demonstrate stronger coordination across systems, yet still lack sufficient capacity to meet demand [13,25].

Across all cities, these system gaps constrain the effectiveness of Housing First and permanent supportive housing, both of which depend on robust service integration to stabilize individuals with high-acuity needs.

### 4.3. Governance Structures Shape Local Capacity and Outcomes

Governance fragmentation is a defining feature of California’s homelessness response, but its impact varies significantly across cities. Los Angeles has the most complex governance environment, with 88 incorporated cities, a county government, and numerous service providers operating within overlapping jurisdictions [1]. Sacramento faces persistent tensions between city and county governments particularly around behavioral health responsibilities, which complicate coordination and accountability [6]. San Diego must navigate relationships among city, county, and regional entities, producing mixed results in implementation [21]. San Jose benefits from strong county leadership but still encounters challenges related to system-wide accountability [18]. Long Beach stands out for its centralized governance and integrated service system [13]. Fresno operates within a simpler governance structure but has limited-service capacity and a political environment that places greater emphasis on enforcement [11,24]. San Francisco maintains centralized governance but experiences political fragmentation and high service costs that shape program design and delivery [30]. Together, these differences influence how cities interpret state mandates, allocate resources, and implement interventions, ultimately shaping local outcomes.

### 4.4. Enforcement Pressures Vary Widely and Reflect Local Political Dynamics

Enforcement-oriented strategies, such as encampment sweeps, anti-camping ordinances, and citation-based policing, are present in every city, but their prominence varies considerably. Fresno relies most heavily on enforcement [11], reflecting limited service capacity and local political priorities. Los Angeles and San Diego face intense public pressure to address visible encampments, leading to periodic enforcement actions despite formal commitments to Housing First [11,21]. San Francisco attempts to balance harm-reduction approaches with episodic enforcement driven by public-health and safety concerns [28]. Sacramento uses Safe Ground sites to manage encampments but continues to employ enforcement as part of its broader strategy [36]. San Jose and Long Beach emphasize outreach and low-barrier shelter options, yet still use enforcement selectively in response to community expectations and public-space concerns [13,23]. Across cities, enforcement does not reduce homelessness and often undermines long-term stability [11], but political pressures make it a persistent feature of local responses.

### 4.5. Prevention Capacity Is Uneven and Strongest in San Jose

Prevention remains the most underdeveloped component of California’s homelessness system, despite strong evidence that targeted financial assistance, legal support, and mediation can significantly reduce inflow [27]. San Jose and Santa Clara County have built the strongest prevention infrastructure in the state, demonstrating measurable success in stabilizing households before they enter homelessness [27]. Los Angeles, San Diego, and San Francisco operate prevention programs but face overwhelming demand that far exceeds available resources [12,21,28]. Sacramento, Fresno, and Long Beach have more limited prevention capacity relative to need [6,13,34], reflecting both funding constraints and competing system pressures. Because inflow often matches or exceeds outflow, prevention remains a critical but underutilized strategy statewide, with substantial implications for long-term system performance.

### 4.6. Interim Housing Models Reflect Local Innovation but Face Structural Limits

Cities across California have developed a wide range of interim-housing models, including Navigation Centers, tiny-home communities, safe-parking programs, bridge shelters, and hotel conversions to address immediate safety and stabilization needs [15,21,26,31]. These models demonstrate substantial local innovation, yet they face common structural constraints that limit their overall impact.

Many sites lack reliable pathways to permanent housing, resulting in longer stays and slower system throughput [6]. Operating costs remain high, particularly for non-congregate models that require intensive staffing and services [29]. Workforce shortages especially in behavioral health and case-management roles further strain program capacity [1,3]. Community opposition continues to shape siting decisions and delay implementation [6], while insufficient behavioral–health integration limits the ability of interim housing to meet the needs of individuals with complex conditions [24]. As a result, interim housing remains necessary but is not sufficient without parallel investments in permanent housing and supportive services.

### 4.7. Distinct Local Patterns Reflect the Interaction of Structure and Governance

Each city’s homelessness system reflects a unique combination of structural pressures and governance dynamics that shape local outcomes.

- Los Angeles: The sheer scale of homelessness magnifies structural barriers, while governance fragmentation limits coordination and slows implementation [1].
- San Diego: Geographic dispersion complicates outreach, even as hotel conversions and interim-housing models show promise [21,22].
- San Jose: Strong county-led prevention infrastructure stands out, yet extreme housing costs continue to constrain rehousing efforts [15,27].
- San Francisco: Service-intensive models deliver high levels of care but cannot overcome severe housing scarcity and high operating costs [29].
- Fresno: Reliance on enforcement reflects limited service capacity and broader regional economic challenges [6,34].
- Sacramento: Rapid population growth and persistent city–county governance tensions shape system performance [6].
- Long Beach: Centralized governance and an integrated service system enhance coordination, even as rising rents create mounting pressure [13].

These differences underscore the need for context-specific strategies rather than one-size-fits-all solutions, highlighting how structure and governance interact to produce distinct local directions. These cross-city patterns provide a foundation for interpreting the broader implications of California’s homelessness environment.

## 5. Discussion

The comparative analysis demonstrates that homelessness in California is shaped by the interaction of structural housing scarcity, behavioral–health system limitations, and governance fragmentation. Although the seven cities operate within the same statewide policy framework, their paths diverge due to differences in housing markets, service capacity, and administrative structures. These findings reinforce the broader literature showing that homelessness is structurally produced and locally mediated rather than attributable to individual-level factors alone.

### 5.1. Structural Constraints and System Capacity

Across all cities, housing scarcity remains the dominant constraint on system performance. Coastal cities face extreme rent burdens and low vacancy rates, while inland regions contend with lower wages, limited supportive-housing capacity, and high poverty rates [4]. These pressures limit the effectiveness of rapid rehousing, vouchers, and interim housing, even when programs are well designed [3,4,24]. The analysis underscores that programmatic interventions cannot compensate for the underlying shortage of deeply affordable and supportive housing.

### 5.2. Behavioral–Health Gaps and Housing First Implementation

Behavioral–health system limitations including insufficient treatment beds, workforce shortages, and fragmented service pathways undermine Housing First implementation across all cities. While service-intensive models in Los Angeles and San Francisco demonstrate strong engagement, their impact is constrained by limited behavioral–health capacity and high operating costs [29]. Inland regions such as Fresno and Sacramento face even more pronounced shortages [1,3]. These findings align with research emphasizing the need for integrated housing and health systems to support individuals with complex needs.

### 5.3. Governance Structures and Local Variation

Governance structures significantly shape local implementation. Fragmented systems such as Los Angeles and Sacramento struggle with coordination and accountability, while more centralized models such as Long Beach demonstrate stronger alignment with Housing First principles [6]. San Jose’s county-led model supports robust prevention infrastructure but complicates performance monitoring [1,13]. These differences help explain why cities with similar structural pressures exhibit different outcomes.

### 5.4. Enforcement Pressures and Political Dynamics

Enforcement-oriented strategies remain common despite evidence that they do not reduce homelessness and may exacerbate instability [11]. Their prominence reflects political pressure rather than evidence-based practice. Fresno relies most heavily on enforcement, while Los Angeles, San Diego, and Sacramento employ it episodically in response to public concern [6,12,21,24]. These patterns highlight the tension between political imperatives and research-supported approaches.

### 5.5. Implications for Statewide Policy

The findings suggest several implications for statewide policy. First, California will need to accelerate the production of deeply affordable and supportive housing to address the structural shortage that underlies system bottlenecks. Expanding behavioral health capacity and integrated care models is equally essential, given the high prevalence of complex needs among people experiencing homelessness. Strengthening governance and accountability mechanisms can help reduce fragmentation and improve coordination across jurisdictions. In addition, prevention should be elevated as a core strategy particularly in high-cost markets where even small financial shocks can precipitate homelessness. Finally, the state and its local partners should deemphasize enforcement in favor of housing- and service-oriented approaches that better support long-term stability.

### 5.6. Contribution to the Literature

This study contributes to the literature by demonstrating how statewide structural pressures manifest differently across local contexts and by identifying governance features associated with more effective system performance. The analysis clarifies the limits of service-intensive models in the absence of structural reforms and highlights prevention as an underdeveloped but essential component of California’s homelessness response. Collectively, these insights underscore the need for coordinated, multi-level strategies that address both immediate service needs and the structural conditions that produce homelessness.

## 6. Conclusions

California’s homelessness crisis reflects the interaction of structural housing scarcity, behavioral–health system limitations, economic inequality, and governance fragmentation. Although the state operates under a unified policy framework, the experiences of its seven largest cities demonstrate that local conditions (housing markets, service capacity, political dynamics, and administrative structures) shape homelessness directions in distinct ways. These findings reinforce research showing that homelessness is structurally produced and sustained by interdependent systems rather than isolated individual-level factors [1,2,3].

Across all cities, the shortage of deeply affordable and supportive housing remains the most significant barrier to reducing homelessness. Even innovative interventions such as Navigation Centers, tiny home communities, hotel conversions, and safe parking programs cannot compensate for the underlying mismatch between housing supply and demand [1,15,21,26,31]. Behavioral–health system gaps further limit the effectiveness of Housing First and permanent supportive housing, particularly for individuals with complex needs. These constraints highlight the need for integrated, systems-level approaches that expand both housing production and behavioral–health capacity.

Governance structures also play a critical role. Cities with more centralized systems, such as Long Beach, demonstrate stronger coordination and more consistent implementation of Housing First principles, while fragmented systems such as Los Angeles and Sacramento struggle with alignment, accountability, and performance monitoring. These differences help explain why cities with similar structural pressures exhibit divergent outcomes.

The analysis also underscores the limits of enforcement-oriented strategies. Although political pressure to address visible encampments is intense, enforcement does not reduce homelessness and may undermine long-term stability by disrupting service connections and increasing legal and financial barriers. Cities that rely heavily on enforcement, such as Fresno, face persistent cycles of displacement without corresponding reductions in homelessness.

Finally, prevention remains an underdeveloped but essential component of California’s homelessness response. San Jose’s Homelessness Prevention System illustrates the potential of targeted financial assistance and legal support to reduce inflow, yet most cities lack the resources or infrastructure to implement prevention at scale. Given that inflow often matches or exceeds outflow, expanding prevention capacity is critical for long-term progress.

Overall, this comparative analysis clarifies how statewide structural pressures interact with local governance and service capacity to shape homelessness outcomes. The findings highlight the need for coordinated, multi-level policy responses that address both immediate needs and the broader structural conditions that produce and sustain homelessness. As California continues to invest in housing, behavioral health, and supportive services, the experiences of these seven cities offer important insights into the possibilities and limitations of local action within a complex statewide system.

## Data Availability

The raw data supporting the conclusions of this article will be made available by the authors on request.
